# Determination of nitrate concentration and its risk assessment in bottled water in Iran

**DOI:** 10.1016/j.dib.2018.06.110

**Published:** 2018-07-02

**Authors:** Mahmood Alimohammadi, Noshin Latifi, Ramin Nabizadeh, Kamyar Yaghmaeian, Amir Hossein Mahvi, Mahmood Yousefi, Peyman Foroohar, Saeedeh Hemmati, Zoha Heidarinejad

**Affiliations:** a Center for Water Quality Research (CWQR), Institute for Environmental Research (IER), Tehran University of medical sciences, Tehran, Iran; b Department of Environmental Health Engineering, School of public health, Tehran University of medical sciences, Tehran, Iran; c Health Equity Research Center (HERC) Tehran University of Medical Sciences, Tehran, Iran; d Iranian Bottled Water Association, Tehran, Iran; e Department of Environmental Health Engineering, Faculty of Health, Hormozgan University of Medical Sciences, Bandar Abbas, Iran

**Keywords:** Bottled water, Risk assessment, Nitrate, Iran

## Abstract

Bottled water is one of the sources of drinking water in many arid and semi-arid countries, including Iran. The greatest concern is the health effects of exposure to excessive nitrate concentrations in drinking water due to the development of methemoglobinemia in children. Therefore, the present study was aimed at determining the concentration of nitrate and its risk assessment in drinking water bottled in Iran. 71 different bottled water brands were identified in this study. The nitrate concentration in water samples was then measured using an Ion Chromatography No. 4110 in accordance Standard Methods for the Examination of Water and Wastewater. The hazard quotient (HQ) of nitrate was calculated using the formula based on input variables including nitrate concentration, water per capita, body weight and reference dose. The results showed that the concentration of nitrate in bottled water was in the range of 0.146–50.1 mg/L (average 10.55 mg/L) in one of which, the concentration of nitrate was higher than the WHO guideline. The mean EDI values for nitrate in different groups of infants, children, teenagers and adults were 0.0795, 0.5633, 0.3976 and 0.3186 mg/kg, respectively. The mean HQ values for nitrate in different groups of infants, children, teenagers and adults were 0.0528, 0.3737, 0.2638 and 0.2114, respectively. In general, the hazard quotient (HQ>1) for the population consuming bottled water, appropriate strategies should be considered in order to reduce the concentration of nitrate in bottled water.

**Specifications Table**

**Table:** 

Subject area	Bottled water quality and risk assessment
More specific subject area	Bottled water nitrate
Type of data	Tables
How data was acquired	Bottled water brands tested were obtained from the Iranian Bottled water association. The nitrate concentration of the samples was measured using an Ion Chromatography No. 4110 in accordance with the method described in “Standard methods: For the examination water and wastewater, 22nd edn”.
Data format	Raw, Analyzed
Experimental factors	71 different brands of high-consumption bottled water in Iran were randomly selected
Experimental features	Determine the concentration level of nitrate
Data source location	Iran
Data accessibility	The data are available with this article

**Value of the data**

• Bottled drinking water is one of the source of nitrate absorption into the body [1], [2], [3], [4], [5]. Non-carcinogenic problems occurred due to the exposure to methemoglobinemia nitrate and syndrome in humans, especially children. Therefore, the risk assessment of nitrate exposure can be helpful in the prevention of methemoglobinemia [6], [7], [8], [9], [10], [11].
• The results of the study indicated that the nitrate concentration in many brands of bottled water is lower than the standard limit, therefore, the consumption of bottled water does not create a threat to the health of consumers.
• Based on risk assessment and data analysis, the highest percentage of HQ>1 was associated with the age group of children, thus the sensitivity should be applied to the selection of drinking water brands for this age group.

## 1 Data

The parameters used to calculate the nitrate risk assessment in bottled water are shown in Table 1. Nitrate concentration and nitrate estimated daily intake (EDI) and hazard quotient (HQ) for the four populations of bottled water consumers have been shown in Table 2.

**Table 1:** Parameters used in the present study for health exposure assessment in drinking water [12], [13], [14], [15], [16].

Parameter	Risk exposure factors	Values for groups				Unit
		Infants	Children	Teenagers	Adults	
Nitrate	*C*_f_	–	–	–	–	mg/L
	*C*_d_	0.08	0.85	2	2.5	L/day
	*B*_w_	10	15	50	78	Kg
	RfD	1.6	1.6	1.6	1.6	mg/kg day

**Table 2:** Nitrate concentration and nitrate estimated daily intake (EDI) and hazard quotient (HQ) for the four populations of bottled water consumers.

Nos.	Nitrateconcentration	EDI				HQ			
		Infants	Children	Teenagers	Adults	Infants	Children	Teenagers	Adults
101	29.96	0.2397	1.6977	1.1984	0.9603	0.1498	1.0611	0.7490	0.6002
102	4.9115	0.0393	0.2783	0.1965	0.1574	0.0246	0.1739	0.1228	0.0984
103	4.0295	0.0322	0.2283	0.1612	0.1292	0.0201	0.1427	0.1007	0.0807
104	9.82	0.0786	0.5565	0.3928	0.3147	0.0491	0.3478	0.2455	0.1967
105	11.37	0.0910	0.6443	0.4548	0.3644	0.0569	0.4027	0.2843	0.2278
106	10.32	0.0826	0.5848	0.4128	0.3308	0.0516	0.3655	0.2580	0.2067
107	27.4105	0.2193	1.5533	1.0964	0.8785	0.1371	0.9708	0.6853	0.5491
108	3.495	0.0280	0.1981	0.1398	0.1120	0.0175	0.1238	0.0874	0.0700
109	50.1	0.4008	2.8390	2.0040	1.6058	0.2505	1.7744	1.2525	1.0036
110	2.5795	0.0206	0.1462	0.1032	0.0827	0.0129	0.0914	0.0645	0.0517
111	5.534	0.0443	0.3136	0.2214	0.1774	0.0277	0.1960	0.1384	0.1109
112	18.819	0.1506	1.0664	0.7528	0.6032	0.0941	0.6665	0.4705	0.3770
113	21.378	0.1710	1.2114	0.8551	0.6852	0.1069	0.7571	0.5345	0.4282
114	8.76	0.0701	0.4964	0.3504	0.2808	0.0438	0.3103	0.2190	0.1755
115	2.64	0.0211	0.1496	0.1056	0.0846	0.0132	0.0935	0.0660	0.0529
116	8.317	0.0665	0.4713	0.3327	0.2666	0.0416	0.2946	0.2079	0.1666
117	19.4665	0.1557	1.1031	0.7787	0.6239	0.0973	0.6894	0.4867	0.3900
118	5.388	0.0431	0.3053	0.2155	0.1727	0.0269	0.1908	0.1347	0.1079
119	2.0635	0.0165	0.1169	0.0825	0.0661	0.0103	0.0731	0.0516	0.0413
120	16.8045	0.1344	0.9523	0.6722	0.5386	0.0840	0.5952	0.4201	0.3366
121	0.519	0.0042	0.0294	0.0208	0.0166	0.0026	0.0184	0.0130	0.0104
122	2.7395	0.0219	0.1552	0.1096	0.0878	0.0137	0.0970	0.0685	0.0549
123	39.4115	0.3153	2.2333	1.5765	1.2632	0.1971	1.3958	0.9853	0.7895
124	17.397	0.1392	0.9858	0.6959	0.5576	0.0870	0.6161	0.4349	0.3485
125	3.1275	0.0250	0.1772	0.1251	0.1002	0.0156	0.1108	0.0782	0.0627
126	7.6265	0.0610	0.4322	0.3051	0.2444	0.0381	0.2701	0.1907	0.1528
127	4.3745	0.0350	0.2479	0.1750	0.1402	0.0219	0.1549	0.1094	0.0876
128	9.055	0.0724	0.5131	0.3622	0.2902	0.0453	0.3207	0.2264	0.1814
129	14.28	0.1142	0.8092	0.5712	0.4577	0.0714	0.5058	0.3570	0.2861
130	5.059	0.0405	0.2867	0.2024	0.1621	0.0253	0.1792	0.1265	0.1013
131	10.36	0.0829	0.5871	0.4144	0.3321	0.0518	0.3669	0.2590	0.2075
132	4.5615	0.0365	0.2585	0.1825	0.1462	0.0228	0.1616	0.1140	0.0914
133	30.73	0.2458	1.7414	1.2292	0.9849	0.1537	1.0884	0.7683	0.6156
134	12.791	0.1023	0.7248	0.5116	0.4100	0.0640	0.4530	0.3198	0.2562
135	11.128	0.0890	0.6306	0.4451	0.3567	0.0556	0.3941	0.2782	0.2229
136	27.9615	0.2237	1.5845	1.1185	0.8962	0.1398	0.9903	0.6990	0.5601
137	7.074	0.0566	0.4009	0.2830	0.2267	0.0354	0.2505	0.1769	0.1417
138	9.141	0.0731	0.5180	0.3656	0.2930	0.0457	0.3237	0.2285	0.1831
139	16.8035	0.1344	0.9522	0.6721	0.5386	0.0840	0.5951	0.4201	0.3366
140	17.149	0.1372	0.9718	0.6860	0.5496	0.0857	0.6074	0.4287	0.3435
141	13.44	0.1075	0.7616	0.5376	0.4308	0.0672	0.4760	0.3360	0.2692
142	2.9085	0.0233	0.1648	0.1163	0.0932	0.0145	0.1030	0.0727	0.0583
143	5.3935	0.0431	0.3056	0.2157	0.1729	0.0270	0.1910	0.1348	0.1080
144	5.629	0.0450	0.3190	0.2252	0.1804	0.0281	0.1994	0.1407	0.1128
145	1.079	0.0086	0.0611	0.0432	0.0346	0.0054	0.0382	0.0270	0.0216
146	15.535	0.1243	0.8803	0.6214	0.4979	0.0777	0.5502	0.3884	0.3112
147	4.49	0.0359	0.2544	0.1796	0.1439	0.0225	0.1590	0.1123	0.0899
148	0.146	0.0012	0.0083	0.0058	0.0047	0.0007	0.0052	0.0037	0.0029
149	15.356	0.1228	0.8702	0.6142	0.4922	0.0768	0.5439	0.3839	0.3076
150	24.643	0.1971	1.3964	0.9857	0.7898	0.1232	0.8728	0.6161	0.4936
151	3.197	0.0256	0.1812	0.1279	0.1025	0.0160	0.1132	0.0799	0.0640
152	12.69	0.1015	0.7191	0.5076	0.4067	0.0635	0.4494	0.3173	0.2542
153	6.6	0.0528	0.3740	0.2640	0.2115	0.0330	0.2338	0.1650	0.1322
154	14.101	0.1128	0.7991	0.5640	0.4520	0.0705	0.4994	0.3525	0.2825
155	7.784	0.0623	0.4411	0.3114	0.2495	0.0389	0.2757	0.1946	0.1559
156	3.345	0.0268	0.1896	0.1338	0.1072	0.0167	0.1185	0.0836	0.0670
157	8.932	0.0715	0.5061	0.3573	0.2863	0.0447	0.3163	0.2233	0.1789
158	32.378	0.2590	1.8348	1.2951	1.0378	0.1619	1.1467	0.8095	0.6486
159	5.468	0.0437	0.3099	0.2187	0.1753	0.0273	0.1937	0.1367	0.1095
160	3.548	0.0284	0.2011	0.1419	0.1137	0.0177	0.1257	0.0887	0.0711
161	16.601	0.1328	0.9407	0.6640	0.5321	0.0830	0.5880	0.4150	0.3326
162	2.643	0.0211	0.1498	0.1057	0.0847	0.0132	0.0936	0.0661	0.0529
163	7.541	0.0603	0.4273	0.3016	0.2417	0.0377	0.2671	0.1885	0.1511
164	7.609	0.0609	0.4312	0.3044	0.2439	0.0380	0.2695	0.1902	0.1524
165	7.095	0.0568	0.4021	0.2838	0.2274	0.0355	0.2513	0.1774	0.1421
166	0.319	0.0026	0.0181	0.0128	0.0102	0.0016	0.0113	0.0080	0.0064
167	1.294	0.0104	0.0733	0.0518	0.0415	0.0065	0.0458	0.0324	0.0259
168	0.428	0.0034	0.0243	0.0171	0.0137	0.0021	0.0152	0.0107	0.0086
169	1.582	0.0127	0.0896	0.0633	0.0507	0.0079	0.0560	0.0396	0.0317
170	1.014	0.0081	0.0575	0.0406	0.0325	0.0051	0.0359	0.0254	0.0203
171	1.97	0.0158	0.1116	0.0788	0.0631	0.0099	0.0698	0.0493	0.0395
Min	0.146	0.0012	0.0083	0.0058	0.0047	0.0007	0.0052	0.0037	0.0029
Max	50.1	0.4008	2.8390	2.0040	1.6058	0.2505	1.7744	1.2525	1.0036
Mean	10.55	0.0844	0.5980	0.4221	0.3382	0.0528	0.3737	0.2638	0.2114
SD	9.94	0.0795	0.5633	0.3976	0.3186	0.0497	0.3520	0.2485	0.1991

## 2 Experimental design, materials and methods

In this study, 71 different brands of high-consumption bottled water in Iran were randomly selected, the nitrate concentration of all bottled water samples was measured using an Ion Chromatography No. 4110 in accordance with the method described in the book “Standard methods: For the examination water and wastewater, 22nd edn” [17], [18], [19], [20], [21], [22]. Then, the health risks of exposure to nitrate in bottled water were assessed according to the classification conducted by Yousefi et al. [12]. for different age groups, which is determined by calculating the hazard quotient (to show non-carcinogenic effects) for different age groups based on the following equations. HQ values less than 1 indicate a non-cancerous effect of the population exposed to exposure. While, if this value is greater than 1, it indicates the occurrence probability of non-carcinogenic effects in the exposed population.

First, the daily nitrate consumption in bottled water was estimated according to Eq. (1) [12], [14]:

(1) $$\mathrm{EDI}=\frac{{C_{f}\times C}_{d}}{B_{w}}$$

EDI: Estimation of daily nitrate consumption
*C*_f_: Nitrate concentration in drinking water
*C*_d_: Average daily drinking water intake
*B*_w_: body weight

Then, HQ risk contribution was calculated to predict non-carcinogenic risk of exposure to nitrate using Eq. (2):

(2) $$\mathrm{HQ}=\frac{\mathrm{EDI}}{\mathrm{RFD}}$$

where, HQ is the risk of non-carcinogenic substances

EDI: Estimated Daily intake (mg/kg d)
RFD: Reference dose
The reference dose for nitrate is 1.6 mg/kg d [14]
